# Maximizing gain in high-throughput screening using conformal prediction

**DOI:** 10.1186/s13321-018-0260-4

**Published:** 2018-02-21

**Authors:** Fredrik Svensson, Avid M. Afzal, Ulf Norinder, Andreas Bender

**Affiliations:** 1 Department of Chemistry, Centre for Molecular Informatics, University of Cambridge, Lensfield Road, Cambridge, CB2 1EW UK; 2 IOTA Pharmaceuticals, St Johns Innovation Centre, Cowley Road, Cambridge, CB4 0WS UK; 3 Unit of Toxicology Sciences, Karolinska Institutet, Swetox, Forskargatan 20, 151 36 Södertälje, Sweden; 4 Department of Computer and Systems Sciences, Stockholm University, Box 7003, 164 07 Kista, Sweden

**Keywords:** Conformal prediction, HTS, Gain-cost function, PubChem datasets

## Abstract

Iterative screening has emerged as a promising approach to increase the efficiency of screening campaigns compared to traditional high throughput approaches. By learning from a subset of the compound library, inferences on what compounds to screen next can be made by predictive models, resulting in more efficient screening. One way to evaluate screening is to consider the cost of screening compared to the gain associated with finding an active compound. In this work, we introduce a conformal predictor coupled with a gain-cost function with the aim to maximise gain in iterative screening. Using this setup we were able to show that by evaluating the predictions on the training data, very accurate predictions on what settings will produce the highest gain on the test data can be made. We evaluate the approach on 12 bioactivity datasets from PubChem training the models using 20% of the data. Depending on the settings of the gain-cost function, the settings generating the maximum gain were accurately identified in 8–10 out of the 12 datasets. Broadly, our approach can predict what strategy generates the highest gain based on the results of the cost-gain evaluation: to screen the compounds predicted to be active, to screen all the remaining data, or not to screen any additional compounds. When the algorithm indicates that the predicted active compounds should be screened, our approach also indicates what confidence level to apply in order to maximize gain. Hence, our approach facilitates decision-making and allocation of the resources where they deliver the most value by indicating in advance the likely outcome of a screening campaign.

**Electronic supplementary material:**

The online version of this article (10.1186/s13321-018-0260-4) contains supplementary material, which is available to authorized users.

## Background

High throughput screening (HTS) has long been a paradigm in early-stage drug discovery [1]. With the advancements in screening technology and automation, it has become feasible to screen libraries in an iterative fashion, screening a small part of the library and using the result to make inferences about what compounds to screen next [2–5]. This allows for a smaller part of the library to be screened while still identifying a large portion of the active compounds. This is a setup that is well suited for machine learning approaches as the first part of the library that is screened can be used to train the learning algorithms.

To evaluate such a machine learning system, we need some way to quantify its performance. Evaluation of virtual screening methods has been the objective of many studies, but tend to focus on how well techniques perform on average across different datasets, often in the form of dedicated benchmark datasets [6, 7]. These evaluations are generally based on how well active compounds are enriched in a certain fraction of the dataset, sometimes with the additional consideration that hits should appear as early as possible in the hit list [8]. However, in an iterative screening scenario, when data from the first screening iteration is available, there are a number of practical considerations of a somewhat different nature, such as how large a portion of the database should be screened in the next iteration, that are not answered directly by the enrichment and related metrics. Consider for example a very small selection of the library yielding a very high enrichment but few identified actives compared to a larger selection of the compound library yielding a lower enrichment but more different chemotypes.

One way to evaluate what number of compounds to screen is to consider the problem in terms of gain and cost, similar to many problems in other fields [9–11]. The evaluation of a compound is associated with a certain cost while the identification of an active compound represents a gain. It is desirable to find a way to select compounds for evaluation in a way that maximizes the overall gain after deducting the cost of screening. This can easily be expressed in a simple function that can be used to evaluate the outcome of any screening set. The main challenge with such an approach is the assignment of the gain component of the gain-cost function. Whereas cost is typically readily assessed, the gain of finding a hit represents a more abstract value. Many different approaches could potentially be factored into the assignment of the gain, for example, one could consider how many compounds it would be acceptable to screen to identify one hit and assign the gain accordingly, or as used in this study, assign the gain to a magnitude that would make a full HTS screen approximately breakeven.

A prerequisite for the gain-cost evaluation to be prospectively meaningful as a tool for evaluating different predictors, is that the results on the training data also extend to new data. Conformal prediction is a framework for generating confidence predictors that produce predictions with a fixed error rate [12]. This is achieved through evaluating new predictions by comparing them to the predictions of known instances in a calibration set. For binary classification, labels are then assigned to the new instance in a way that can result in four different outcomes: the instance belongs to either of the two labels, both labels simultaneously or none of the labels. Two factors make conformal predictors highly suitable for bioactivity prediction: their ability to accurately predict minority classes [13–15], since in a screen there tends to be many inactive compounds for each active, and the ability to control the error rate and thereby limiting the number of false positives. Conformal predictors have previously been successfully applied for bioactivity modelling [3, 16, 17].

As the efficiency (number of single label predictions) generated by the conformal predictor will vary depending on the confidence level applied, evaluating different confidence levels will identify if it is better to screen a small set of compounds with higher confidence or a larger set but with more uncertainty. This approach also does not require the user to decide on an exact number of compounds to screen in the next iteration, but instead, this will be provided by the predictor based on the selected confidence. For the conformal prediction framework to guarantee the error rate, the data considered needs to be exchangeable [12]. In an iterative screening setup, this has implications on how to select the compounds for the initial round of screening, and the only way to guarantee exchangeability (as long as the sample is large enough) would be to draw a random sample of the available data.

We have previously reported a preliminary study on the use of a combination of a gain-cost function and conformal predictors in the context of iterative screening [18]. In this work, we expand this to more datasets and provide a more in depth analysis. By training conformal predictors on an initial training set consisting of 20% of each dataset, we show that the parameters that optimise gain in the remaining screening set can be identified. Overall, this study shows that this conformal gain-cost driven method is a promising approach to optimize compound selection in screening programs in terms of optimising the gain.

## Methods

### Data

Large screening datasets were selected from PubChem [19] to represent a spread in terms of size and ratio of active to inactive compounds. 12 selected datasets (Table 1) were downloaded from PubChem and prepared using the IMI eTOX project standardizer [20] in order to generate consistent compound representations. The structures were then further subjected to tautomer standardization using the MolVS standardizer [21]. Activity was assigned according to the PubChem annotation, and compounds with ambiguous activity were discarded.

**Table 1:** The datasets employed in this study

AID	Description	Active	Inactive	% Active
411	qHTS Assay for Inhibitors of Firefly Luciferase	1577	70,097	2.2
868	Screen for Chemicals that Inhibit the RAM Network	3545	191,037	1.8
1030	qHTS Assay for Inhibitors of Aldehyde Dehydrogenase 1 (ALDH1A1)	16,117	148,322	7.8
1460	qHTS for Inhibitors of Tau Fibril Formation, Thioflavin T Binding	5825	221,867	2.6
1721	qHTS Assay for Inhibitors of Leishmania Mexicana Pyruvate Kinase (LmPK)	1089	290,104	0.4
2314	Cycloheximide Counterscreen for Small Molecule Inhibitors of Shiga Toxin	37,055	259,401	12.5
2326	qHTS Assay for Inhibitors of Influenza NS1 Protein Function	1073	260,701	0.4
2451	qHTS Assay for Inhibitors of Fructose-1,6-bisphosphate Aldolase from Giardia Lamblia	2061	276,158	0.7
2551	qHTS for inhibitors of ROR gamma transcriptional activity	16,824	256,777	6.1
485290	qHTS Assay for Inhibitors of Tyrosyl-DNA Phosphodiesterase (TDP1)	986	345,663	0.3
485314	qHTS Assay for Inhibitors of DNA Polymerase Beta	4522	315,791	1.4
504444	Nrf2 qHTS screen for inhibitors	7472	285,618	2.5

### Feature generation

We have previously applied a set of 97 physicochemical/structural feature descriptors in previous studies with good results [3, 15]. These 97 descriptors (physicochemical), as well as full-length Morgan fingerprint descriptors (fingerprints), were calculated using RDKit [22]. The latter were subsequently hashed onto a binary feature vector of length 4096 by modulo calculations on the generated fingerprint indices using an in-house Perl script.

### Machine learning and conformal prediction

**Fig. 1:**
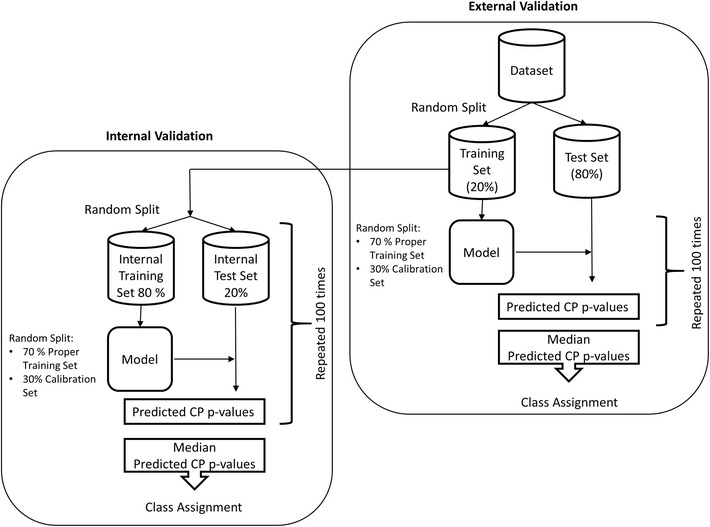
Schematic representation of the validation procedure used in this study

Each dataset was split into an initial screening or training set (20%) and a test set (80%). The number of active and inactive compounds in the training and test set after processing is shown in Table 2. Internal model validation was performed by randomly splitting the training data into an internal training (80%) and test (20%) sets which were resampled prior to every model building. The training data for building the models was further randomly split into a proper training (70%) and calibration set (30%). This random split was also re-performed prior to building every model. The data-split and validation strategy is shown schematically in Fig. 1.

**Table 2:** Number of compounds in training and test data for all the datasets after data processing

AID	Train active	Train inactive	Test active	Test inactive
411	340	13,761	1215	55,187
868	326	19,129	3219	171,705
1030	3240	29,090	12,674	116,642
1460	132	4637	1057	41,197
1721	219	57,905	868	231,624
2314	3730	25,769	33,225	232,103
2326	190	51,988	877	207,835
2451	422	54,560	1594	218,333
2551	1681	25,443	14,951	227,744
485290	192	67,593	761	270,377
485314	857	62,561	3634	250,038
504444	1524	56,628	5882	226,723

All models were developed using scikit-learn [23], using default parameters unless otherwise indicated, and inductive conformal predictors were derived utilising the nonconformist package [24]. For all models random forest ensembles consisting of 500 trees were used as the underlying models. We applied the aggregated conformal predictor procedure using 100 iterations [25]. For internal validation, each one of these iterations randomly leaves out 20% of the compounds and the generated model is used to predict the left out compounds. The median of the predicted conformal prediction p-values for each class (active or inactive) across all iterations is then used to derive the predicted labels.

**Fig. 2:**
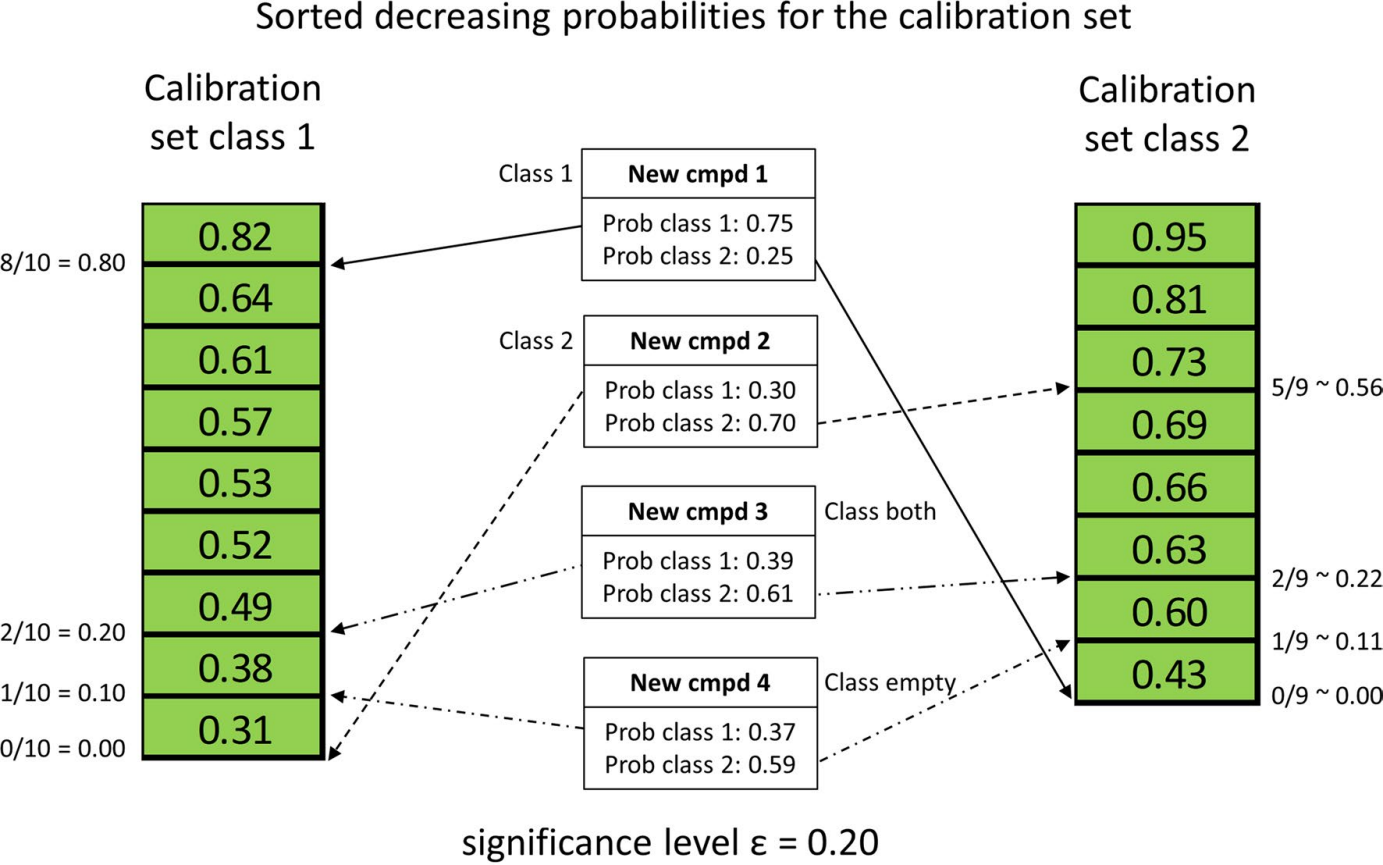
Illustration of how conformal prediction classes are assigned

Using the percentage of trees in the random forest ensemble predicting each of the classes (class probability) as the conformal prediction conformity (similarity) measure the method assigns classes to new compounds by comparing the class probability against the corresponding sorted list for the calibration set, see Fig. 2.

The predicted class probabilities for classes 1 and 2, e.g. active and inactive class, of the new compound is placed in the sorted list of the calibration set probabilities for classes 1 and 2, respectively, and thus adding one compound to the list for each class. For each class, the position of the new compound in these sorted lists is determined and the fraction with lower probabilities is calculated. This fraction is, for each class, compared to the corresponding significance level set by the user. For a new compound to be part of a class the computed fraction must be larger or equal to the set significance level.

This procedure is illustrated for the four possible outcomes from a binary classification task in Fig. 2. New compound 1 has predicted class probabilities for class 1 and 2 of 0.75 and 0.25, respectively. Placing these probabilities in the corresponding sorted calibration set list of probabilities results in positions 9 and 1, respectively, and the corresponding calculated fractions are 0.80 and 0.0. The set significance level in this example is 0.20. This means that new compound 1 can be assigned to class 1 (0.80 ≥ 0.20) but not to class 2 (0.0 < 0.20). Similarly, new compound 2 can only be assigned to class 2. However, for new compound 3 and 4 the situation is different. For new compound 3 the calculated fractions for both classes are above or equal to the set significance level and, consequently, this compound is assigned to both class 1 and 2 (the “both” class). For new compound 4 the situation is the opposite and both calculated fractions are below the set significance level. Thus, new compound 4 cannot be assigned to any of the two classes by the model (the “empty” class). For new compound 4 it should be noted, for clarity, that 4 decision trees did not give a class assignment, e.g. the resulting leaf node was unable to provide a majority class vote.

For a more in-depth explanation of the implementation of conformal prediction, we refer the reader to a recent study by Norinder et al. [26].

### Gain-cost function

As previously described [18], we defined a gain-cost function to evaluate the results from the screening

1 $$gain = \mathop \sum \limits_{i = 1}^{{\left| {train} \right|}} hit\,gain - \mathop \sum \limits_{i = 1}^{{\left| {train} \right|}} screen\,cost + \mathop \sum \limits_{i = 1}^{{\left| {test} \right|}} hit\,gain - \mathop \sum \limits_{i = 1}^{{\left| {test} \right|}} screen\,cost.$$

We applied three different screening cost levels (arbitrary units), high (14), medium (10), and low (6). The different cost levels can be thought of as representations of different assay setups, where for example a more complex phenotypic assay is more costly per compound screened compared to a biochemical assay on isolated protein [27, 28]. We then decided on a gain of 400 per identified hit. These values were applied in our previous study on gain-cost [18], and represent a gain-cost balance that, on average, would result in an approximately breakeven outcome, in terms of cost-gain, for the four HTS screening campaigns considered in that study.

### Summary of screening set-up

The screening workflow proposed in this study utilizes an initial screen of 20% of each compound library. The results from this initial screening are then used to train a conformal predictor and different confidence levels of the predictor are then evaluated using the internal validation procedure and the defined gain-cost function. High confidence levels will generate few predicted active compounds with a higher accuracy while a low confidence level will generate many predicted actives with lower accuracy. This way it is evaluated if it is better to screen many compounds expecting a lower hit-rate or few compounds with a higher hit-rate. All the initial screening data (20% of each library) was then used to construct a predictor that was used to predict the remaining 80% of the screening library based on the confidence level indicated from the internal validation to give the highest gain. Compounds receiving a single label prediction as active are then considered for testing.

### Performance evaluation measures

Since the prediction of a conformal predictor is a set of labels rather than always a single label, they are generally evaluated by their *validity* and *efficiency* [12]. Validity is defined as the fraction of predictions containing the correct label. This means in a binary classification that a single label prediction is correct if the label is the correct one, a dual label is always correct, and an empty prediction is always incorrect. The validity is guaranteed to correspond to the user-defined confidence level as long as the data is exchangeable. The efficiency of a conformal predictor is defined as the number of single label predictions, a higher fraction of single label predictions means a more efficient predictor.

## Results and discussion

Table 3 summarizes the validities of the generated conformal predictors. Overall the models based on physicochemical descriptors corresponded better to the set confidence level which is of importance in conformal prediction in relation to what can be expected from predictions on new data [12]. We therefore choose to base the main discussions around the results from the physicochemical-based models while supplementing the discussion with results from the fingerprint based models when merited. Detailed results for both approaches is available in the Additional file 1.

**Table 3:** Average validity of the physicochemical and fingerprint based models

	Confidence level			
	90%	80%	70%	60%
*Physiochemical*				
Validity train active	0.928	0.833	0.728	0.631
Validity train inactive	0.910	0.813	0.715	0.614
Validity test active	0.922	0.818	0.718	0.615
Validity test inactive	0.907	0.811	0.714	0.615
*Fingerprint*				
Validity train active	0.976	0.896	0.771	0.627
Validity train inactive	0.949	0.888	0.809	0.694
Validity test active	0.972	0.895	0.766	0.610
Validity test inactive	0.943	0.884	0.810	0.714

An overview of the performance of the models using the physicochemical descriptors is summarised in Table 4. It can be seen that the resulting models for the different datasets varied greatly in performance. Some datasets were poorly predicted, especially the two datasets 2326 and 485290 produced poor models with very low efficiency (0.395 and 0.51 respectively), likely due to the extreme imbalance in the ratio of active to inactive compounds, 0.37 and 0.28%, respectively (Table 2), in the training data. The other datasets showed satisfactory outcomes with validities close to the desired 0.8 and efficiencies ranging from 0.6 to 0.9 in the internal validations on the training data. The trends observed in the training data when applying the internal validation procedure translated very well to how the models performed when applied to the test data with an average absolute difference in the validity of 0.02 and 0.01 for active and inactive compounds respectively.

**Table 4:** Validity and efficiency for active and inactive compounds at the 80% confidence level for the derived conformal predictors based on physicochemical descriptors

AID	Validity active	Efficiency active	Validity inactive	Efficiency inactive
411 train	0.856	0.809	0.815	0.771
411 test	0.873	0.847	0.811	0.794
868 train	0.828	0.798	0.813	0.835
868 test	0.825	0.844	0.805	0.862
1030 train	0.823	0.654	0.819	0.636
1030 test	0.832	0.677	0.807	0.653
1460 train	0.864	0.864	0.816	0.88
1460 test	0.748	0.944	0.805	0.957
1721 train	0.868	0.918	0.842	0.899
1721 test	0.869	0.933	0.835	0.907
2314 train	0.813	0.81	0.807	0.808
2314 test	0.801	0.833	0.803	0.819
2326 train	1	0.395	0.856	0.144
2326 test	1	0.511	0.849	0.151
2451 train	0.884	0.746	0.836	0.66
2451 test	0.859	0.778	0.828	0.707
2551 train	0.819	0.916	0.809	0.906
2551 test	0.812	0.944	0.803	0.934
485290 train	1	0.51	0.86	0.15
485290 test	1	0.545	0.863	0.137
485314 train	0.846	0.762	0.824	0.726
485314 test	0.856	0.799	0.818	0.743
504444 train	0.833	0.749	0.813	0.755
504444 test	0.818	0.767	0.811	0.771

Train denotes the results from the internal validation and test when the models are applied to the external test set

**Fig. 3:**
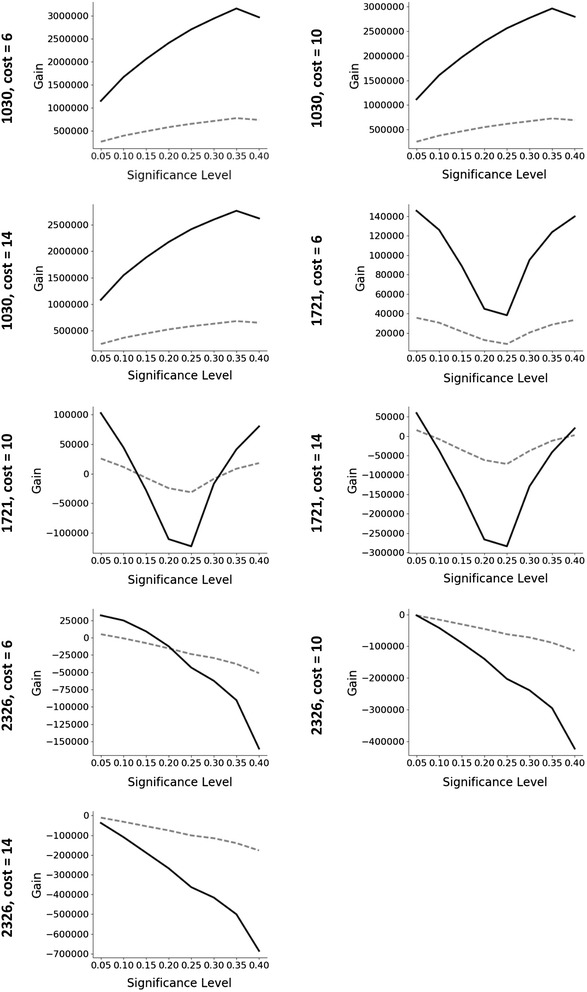
Evaluation of the gain-cost function for three examples showing different trends (using the physicochemical based descriptors models). The dashed line represents test data and the solid line evaluation of the remaining data. Trends observed in the training data generally predict the trend on the remaining test data very well

The total gain-cost evaluation for both the internal validation and the remaining test dataset for three cases displaying very different trends are shown in Fig. 3 (plots for all the datasets are available in the Additional file 1). Although the outcome is different between the datasets, it can be seen that the trend observed on the gain-cost evaluation on the training data is closely mirrored also in the test data.

If the optimal approach identified using the internal validation procedure had been used to select the next screening set from the remaining compounds, the gain would have been maximized in 8 of the 12 datasets at screening cost 6, in 9 of the 12 datasets at screening cost 10, and in 10 of the 12 datasets at screening cost 14 (see Additional file 2 for tabularised values).

Three principally different outcomes from the results of the cost-gain evaluations were indicated by the internal validation procedure: to screen the compounds predicted to be active (maximum gain obtained for one of the evaluated conformal predictors), to screen all the remaining data (maximum gain obtained for the full training data), or not to screen any additional compounds (all screening outcomes indicate a loss).

Furthermore, for the cases where the maximum gain for the test set was not correctly predicted by the internal training set validation, e.g. 411, 868, 1460, 2326, 2451 and 485314 at various cost levels, the average loss percentage of the total gain (training and test set) is, with one exception, very small and only in the order of 0.5–2.1% (Table 5). For dataset 2326 using fingerprints, the internal validation significantly underestimates the subsequent total gain by as much as 24%. This is one of the more poorly modelled datasets, potentially indicating that this approach should not be attempted if the internal validation of the models indicates poor performance.

**Table 5:** Average percent loss in gain where training data did not correctly predict maximum gain for the test set

Cost	Total number of partially screened datasets^a^	Fingerprint based models		Physiochemical based models	
		Number of dataset^b^	%loss	Number of dataset^b^	%loss
6	9	6	5.7^c^	4	2.1
10	10	3	1	3	1.8
14	10	3	1.6	2	0.4

^a^Datasets where the validation did not indicate that the entire set should be screened for maximum gain

^b^Datasets where the optimum training set validation setting did not correspond to the maximum test set gain

^c^Fails for dataset 2326: 23.9%. Excluding this result: 2.1%

Despite having a generally lower performance with regards to validity and efficiency, the models based on fingerprints seem to be able to identify settings for the confidence levels that enable somewhat higher gains from screening the training set and the predicted test subset gain, compared to the corresponding models based on physicochemical descriptors for the investigated datasets (Table 6). However, it is difficult to compare the performance in terms of percentage since in some cases, 485314 at cost level 14, will generate a loss for the physicochemical descriptor-based model in comparison to a small gain for the fingerprint based model. Excluding this example the fingerprint models perform, on average, 14–16% better with large variations (2–51%) between datasets and cost levels. On the other hand, for dataset 868, where the physicochemical descriptor-based model outperforms the fingerprint-based model, the improvement is 23, 42 and 71% for cost levels 6, 10 and 14, respectively. Considering the grave underestimation of dataset 2326 (Table 5), the latter models seem to be more robust in nature compared to the corresponding models based on fingerprints.

**Table 6:** Number of times the highest gain (training and test set) was obtained from fingerprint (FP) and physicochemical (PC) descriptors based models respectively

Cost	Max gain FP	Max gain PC	Ties^a^
6	6	3	3
10	9	1	2
14	9	1	2

^a^Ties occur when the validation indicates that the entire library should be screened

Another important aspect of the presented procedure in this work is the correct identification of the cases where it would be beneficial, from a gain perspective, to screen the entire library as opposed to a subset of the same library. For datasets 1030, 2314 and 2551 the predictions from the internal validation indicate that screening the entire library would result in the highest gain. This is subsequently also found in all cases for screening the corresponding test set. Also for all cases where the training set validation procedure indicates that no gain can be obtained for the screening, this translated to the corresponding test set.

For some of the investigated cases the internal validation indicates a gain for screening a subset, but when considering the cost for screening the full training set (in order to build the predictive models) the result is an overall loss. This is strongly correlated to the percentage of active compounds in the training set. Thus, the investigated datasets with fewest actives, i.e. 1721, 2326 and 485290, show this behavior for many of the cost levels. For the 19 cases where the cost of screening the full training set is more than five times the indicated gain for screening a subset of the training set, only in one case (dataset 868, fingerprints, cost 14, factor 8) does the subsequent subset screening of the test set result in a small overall gain. This is an additional factor to consider when deciding to screen a particular compound library for a target, namely, that if the cost of screening in relation to the predicted gain is very large, as indicated by the internal validation of the small training set, then the likelihood of generating an overall gain from subsequent screening of a predicted subset is very low. Again, such indications add knowledge for deciding upon to perform a subsequent screen or not of the remaining library for the target in question.

The high translatability of the results from internal model validation is a key feature of the presented approach. Knowing in advance what the likely outcome of a screening campaign will be in terms of gain facilitates decision making and allow resources to be focused where testing delivers the most value. However, the results from this kind of evaluations are only one factor and other aspects, e.g. importance of the target in question and/or finding new (types of) active compounds, will also influence decisions and may also be taken into consideration.

Although we applied the cost-gain function together with a conformal predictor, the concept of a cost-gain functions as a way to evaluate compound selection for screening can be paired with any prediction method. In our opinion this has many advantages over traditionally used evaluation metrics and we hope that this approach will be more widely applied than just within the context described herein.

Nevertheless, we think the conformal framework adds a number of additional benefits. Firstly, it provides an easy way to tune the number of single class predictions. Secondly, the setup is in our opinions easier to communicate to non-computational scientists since the concept of certainty is easily appreciated. Finally, conformal predictors are an easy way to handle the imbalance in the datasets used for training, where there are typically very few active compounds compared to inactive.The presented cost-gain function in this work represents a very basic approach and additional research is required both to establish how to best assign the gain component as well as expanding the complexity of the considered variables. These questions and the expansion to more datasets will be the focus of future studies.

## Conclusions

We present a workflow for the optimization of screening gain based on conformal prediction and a gain-cost function. This approach represents a new way of evaluating iterative screening campaigns and optimizing screening efficiency. This approach was applied to 12 bioactivity datasets derived from PubChem using two different feature spaces, and we show that the method consistently indicates the optimal gain or a very close approximation to the optimal gain on the test data.

We show that, by using 20% of the screening library as an initial screening set, very accurate predictions of the gain for the remaining library can be obtained, identifying the optimal gain in between 8 and 10 out of 12 cases depending on the cost function used. Also, when the algorithm fails to identify the optimal settings the loss compared to the maximum is very small, in all but one case, falling in the range of 0.5–2.1%.

The described approach provides guidance on what selection of compounds to screen from the remaining library, or where appropriate, indicates that the entire library or that no more compounds should be screened in order to maximize gain or, for the last case, minimize loss.

## Additional files

**Additional file 1.** Plots showing the results of the gain-cost function for each dataset using three different cost levels.

**Additional file 2.** Information about the applied datasets, performance of the predictive models, and evaluation of the gain- cost function for the different datasets and settings.

## Abbreviation

HTS: high throughput screening
