# Supplementary material for: Maximizing gain in high-throughput screening using conformal prediction
Source: J Cheminform. 2018 Feb 21;10:7. doi: 10.1186/s13321-018-0260-4 (PMC5821614; doi:10.1186/s13321-018-0260-4)
Supplement: Supplementary file 1 — Additional file 1. Plots showing the results of the gain-cost function for each dataset using three different cost levels. [file 13321_2018_260_MOESM1_ESM.docx]

**Supporting Information**

**Maximizing Gain in High-Throughput Screening Using Conformal Prediction**

Fredrik Svensson^†,‡*^, Avid M. Afzal^†^, Ulf Norinder^ꓕ,§^, Andreas Bender^†^

^†^ Centre for Molecular Informatics, Department of Chemistry, University of Cambridge, Lensfield Road, Cambridge CB2 1EW, UK

^‡^ IOTA Pharmaceuticals, St Johns Innovation Centre, Cowley Road, Cambridge CB4 0WS, UK

^ꓕ^Swetox, Karolinska Institutet, Unit of Toxicology Sciences, Forskargatan 20, SE-151 36 Södertälje,

Sweden

^§^ Department of Computer and Systems Sciences, Stockholm University, Box 7003, SE-164 07 Kista, Sweden

*Corresponding author

**Figure S1.** Evaluation of the gain-cost function for each dataset using three different cost levels (6, 10, 14) for the physicochemical based descriptors models. The dashed line represents test data and the solid line evaluation of the remaining data. Trends observed in the training data generally predicts the trend on the remaining test data very well.

| **6** | **10** | **14** |
| --- | --- | --- |
|  | **AID411** |  |
| 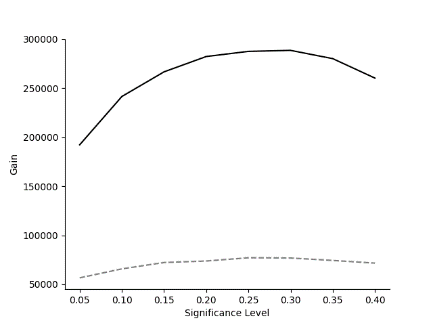 | 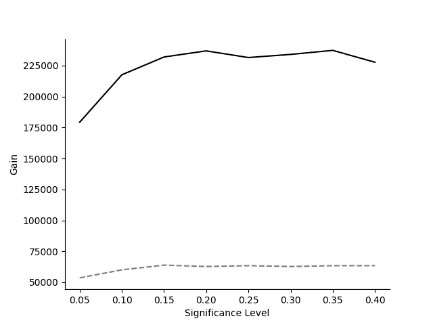 | 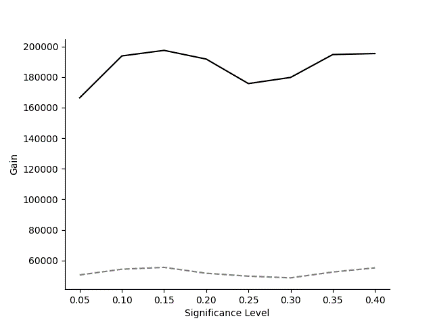 |
|  | **AID868** |  |
| 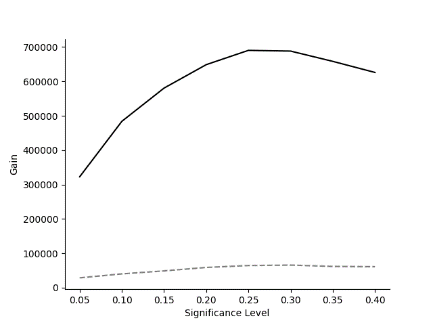 | 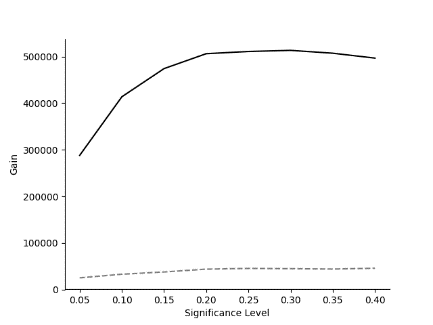 | 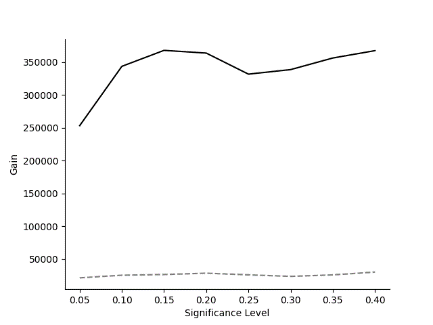 |
|  | **AID1030** |  |
| 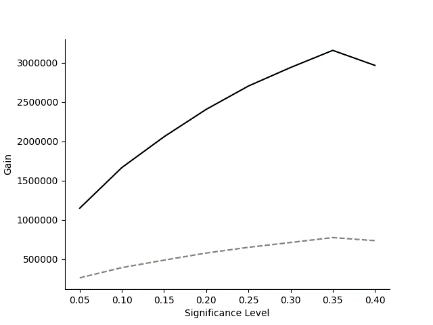 | 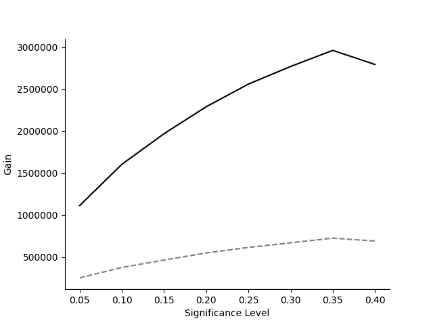 | 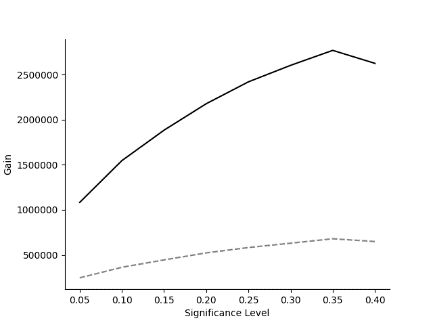 |
|  | **AID1460** |  |
| 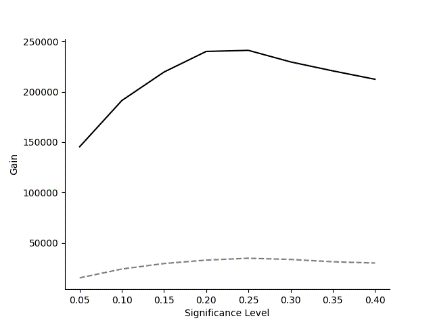 | 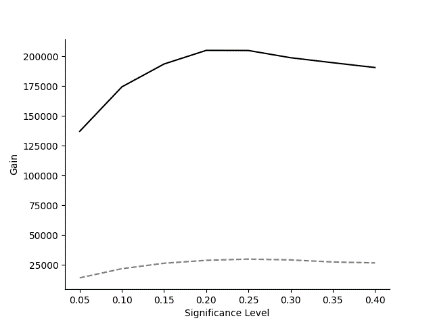 | 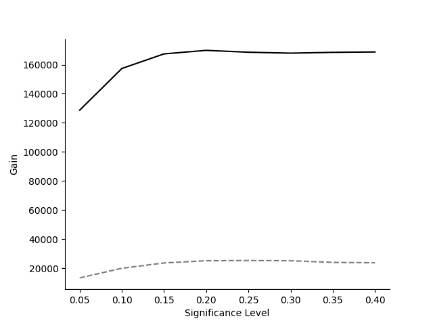 |
|  | **AID1721** |  |
| 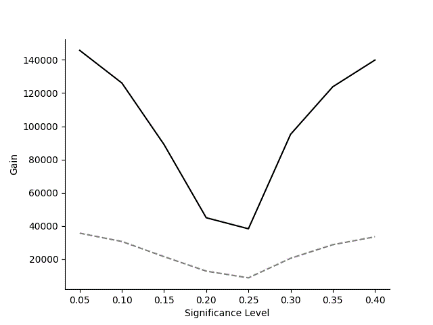 | 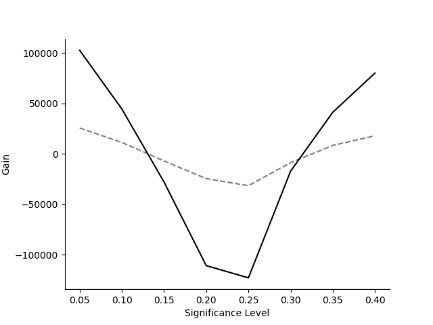 | 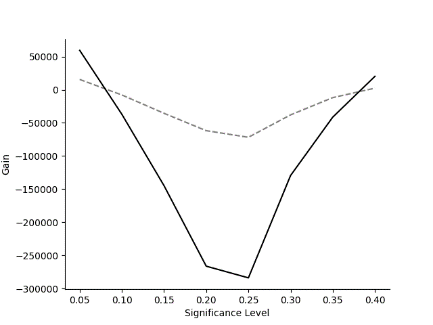 |
|  | **AID2314** |  |
| 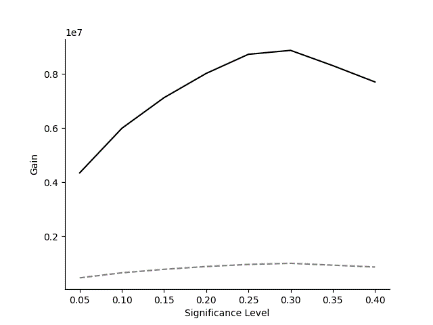 | 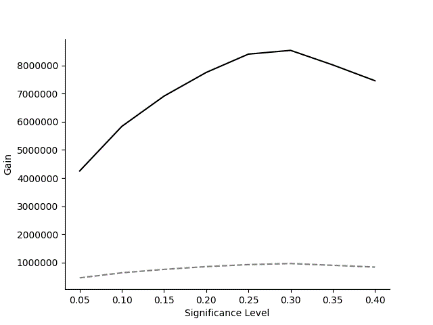 | 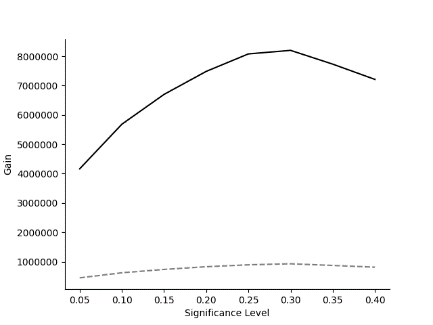 |
|  | **AID2326** |  |
| 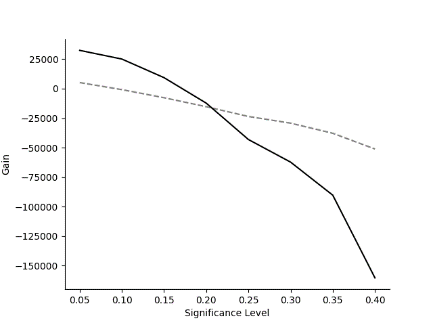 | 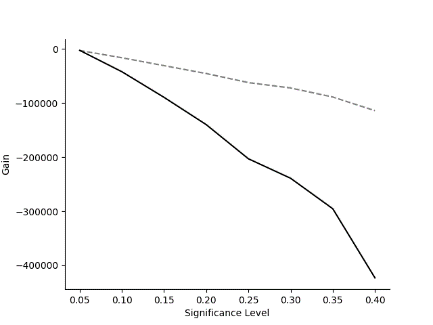 | 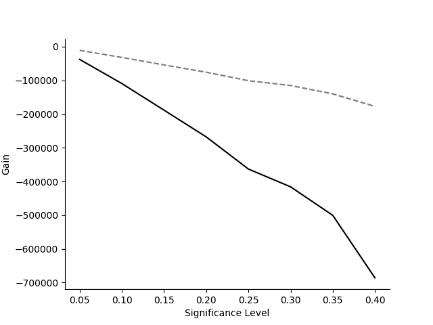 |
|  | **AID2451** |  |
| 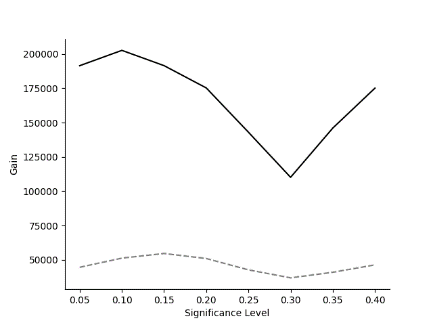 | 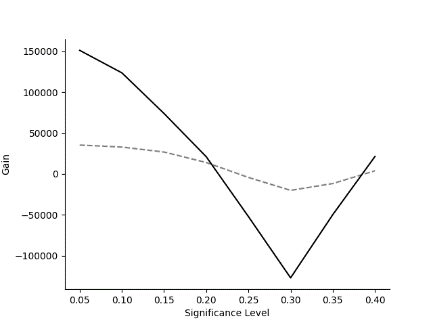 | 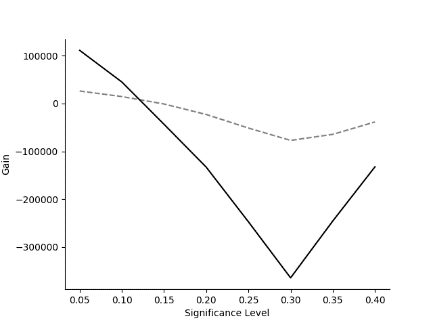 |
|  | **AID2551** |  |
| 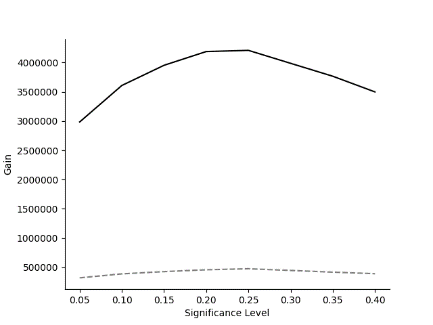 | 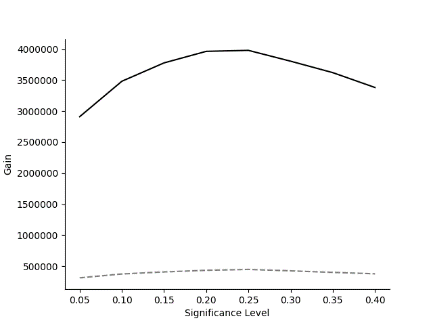 | 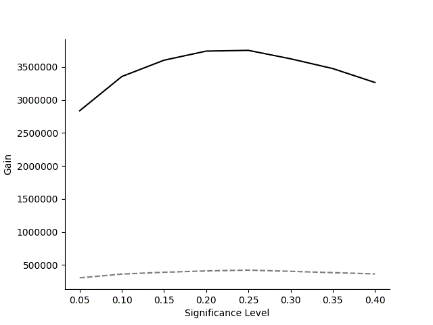 |
|  | **AID485290** |  |
| 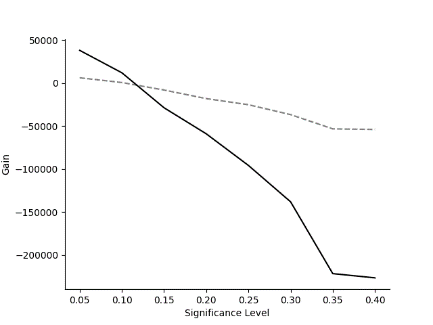 | 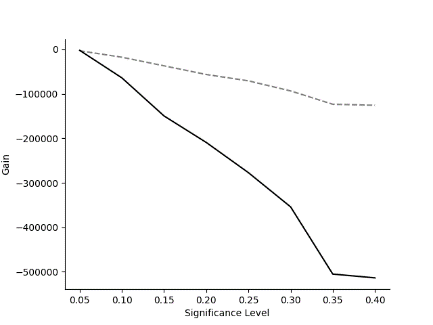 | 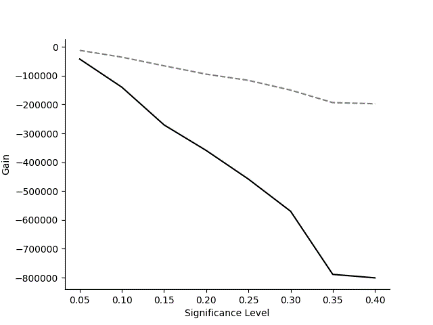 |
|  | **AID485314** |  |
| 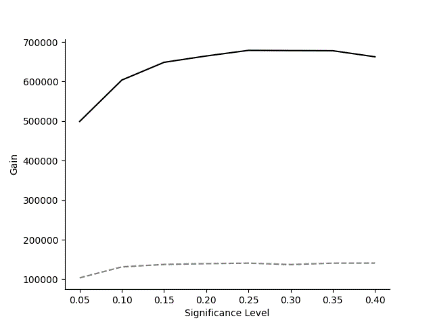 | 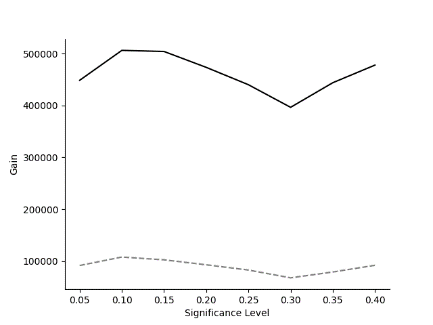 | 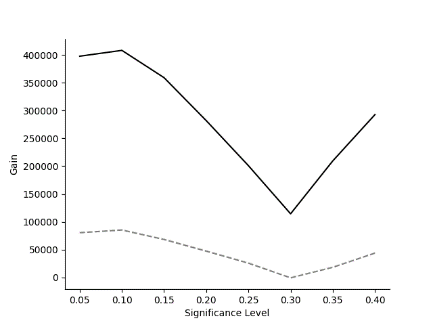 |
|  | **AID504444** |  |
| 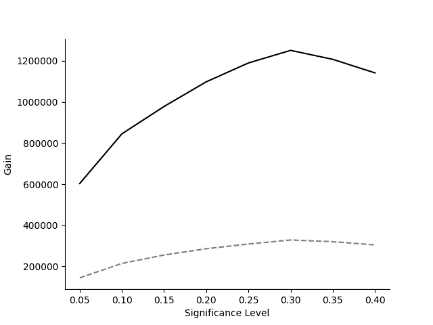 | 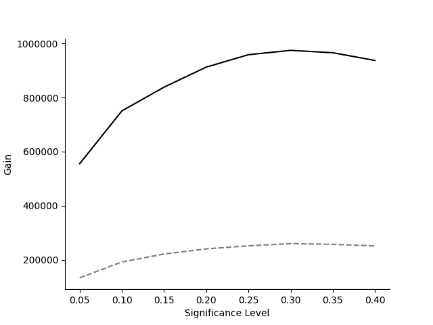 | 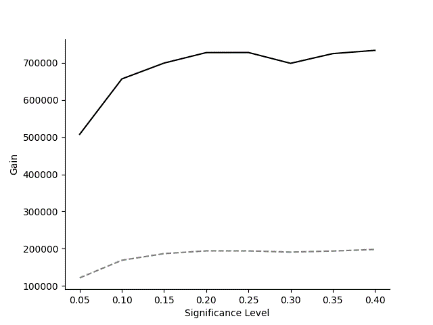 |
